# Effects of Transcranial Direct Current Stimulation in writing: a case report of deep agraphia

**DOI:** 10.1590/2317-1782/20212020319

**Published:** 2022-02-02

**Authors:** Nathani Cristine do Carmo Ramos, Cláudia Aparecida Pietrobon, Ricardo Marcio Garcia Rocha, Luciana Lilian Louzada Martini, Luciano Grüdtner Buratto, Maysa Luchesi Cera

**Affiliations:** 1 Programa de Pós-Graduação em Ciências do Comportamento, Universidade de Brasília – UnB – Brasília (DF), Brasil.; 2 Secretaria de Saúde do Distrito Federal – SES/DF – Brasília (DF), Brasil.; 3 Faculdade de Ceilândia, Universidade de Brasília – UnB – Brasília (DF), Brasil.

**Keywords:** Agraphia, Stroke, Handwriting, Transcranial direct current stimulation, Aphasia

## Abstract

We present the case report of a 61-year-old male participant with chronic conduction aphasia and deep agraphia after ischemic stroke who received training on writing under dictation associated with transcranial direct current stimulation. The treatment consisted of five 50-minute dictation sessions with the application of 2 mA of anodal transcranial direct current stimulation for 20 minutes over the left occipitotemporal cortex. The participant improved his written production of pseudowords and regular low-frequency words, via the phonological route, in addition to a small improvement in the production of irregular words, via the lexical route. After training, there was also a small improvement in writing for untrained stimuli, suggesting generalization. In the assessment carried out 5 months after the end of the treatment, the benefit was maintained for stimuli processed via the phonological route. The results are promising given the severity and chronicity of the case and suggest that transcranial direct current stimulation associated with writing therapy represents a possible clinical alternative for patients with deep agraphia.

## INTRODUCTION

Agraphia is an acquired writing disorder characterized by alterations in spontaneous writing and writing under dictation that cannot be attributed only to perceptual or motor deficits^(1)^. The diagnosis is based on assessing which processes of the written language are altered or preserved^(2)^.

In addition to the application of spontaneous writing tests, written naming writing under dictation, and copying^(2)^, this assessment must encompass different degrees of phonological (e.g., sequence of graphemes) and lexical (e.g., word form) representations. Pseudowords, for example, require preserved phonological processing – via phonological route – to be written correctly^(3)^. In contrast, irregular words demand access to orthographic representations – via lexical route –, since following only the phonological rules can lead to writing errors^(3,4)^.

Individuals with lexical agraphia present alterations in the writing of irregular words and errors of regularization due to the use of phonographemic conversion procedure^(1,2)^. Deficits in the writing of low-frequency words or pseudowords are characteristics of phonological agraphia, whereas deep agraphia is characterized by the occurrence of plenty of errors when using the lexical route, as well as the inability to use the phonological route^(1,2)^.

In neural terms, evidence points to a task division among different cortical areas involved in writing. Phonological processing is associated with a left perisylvian network that includes the superior temporal gyrus, posterior part of the supramarginal gyrus, and Heschl’s gyrus^(5)^. Lexical processing, in turn, is associated with the activity in occipitotemporal areas^(4)^. Focal lesions in areas responsible for the phonological and lexical routes can cause specific writing deficits. For example, lesions in the left occipitotemporal cortex result in disproportionate impairment in the writing of irregular words (lexical route) relative to regular words (lexical and phonological routes) or pseudowords (phonological route)^(1)^. In such cases, rehabilitation can be aimed at improving the semantic and lexical systems and the phonographic conversion route, or both, to reorganize the writing activity^(2)^.

Over the past decade, researchers have explored the association of traditional treatments seeking to favor communication with neuromodulation techniques to maximize their clinical effects ^(6)^. In individuals with post-stroke, Broca’s aphasia, for example, the concomitant use of transcranial direct current stimulation (tDCS) and naming treatments have resulted in improved performance in naming tasks ^(6,7)^.

TDCS is a non-invasive stimulation technique that involves the application of a weak direct electric current (1–2 mA) through two or more electrodes placed on the scalp. The constant electric field resulting from the stimulation influences the electrical activity of the underlying neural tissue^(8)^. In general, the anode (positive electrode) has a stimulatory effect, and the cathode tDCS (negative electrode) has an inhibitory effect on the cortical areas affected^(6)^.

Most of the tDCS studies conducted until now have assessed the performance of individuals with impairments in oral language through the stimulation of areas traditionally associated with speech, such as Broca’s area^(6,7)^. In the very few tDCS studies with individuals with writing deficits, the targets of stimulation are areas associated with the motor aspects of writing (left primary motor cortex in individuals with Parkinson disease)^(9)^ or convergence areas between speech and writing (lower left frontal gyrus in individuals with primary progressive aphasia)^(10)^. None of these studies addressed the stimulation of posterior areas, such as the left occipitotemporal cortex, associated with the phoneme-grapheme conversion process and the lexical access in cognitive neuropsychological models of writing^(4)^.

In this report, we present the case of an elderly individual with deep agraphia who underwent a treatment protocol involving writing under dictation with the concurrent application of tDCS to the left occipitotemporal cortex. The main hypothesis is that the anodal stimulation applied to the left occipitotemporal area will benefit the writing of trained words processed through the phonological and lexical routes.

## CLINICAL CASE PRESENTATION

The participant was a 61-year-old male cook, with 5 years of formal education, who is a former alcoholic, former smoker, currently presenting with controlled type 2 diabetes mellitus and hypertension, chronic renal failure and corrected visual impairment. The participant has a history of communication disorders, conduction aphasia, and deep agraphia after ischemic stroke in the posterior area of the left parietal and temporal lobes.

Six years after his stroke, the patient started rehabilitation with weekly traditional speech therapy. During the initial treatment, emission skills and listening and writing comprehension were stimulated. Six months after the weekly traditional treatment, the participant presented significant functional improvement of oral emission. During that period, the participant no longer attended the treatment as he felt satisfied with the gains in his oral communication, which was his main objective. However, his writing was still impaired. Aiming to promote improvement in the performance of written language, we proposed that he participated in intensive writing treatment associated with tDCS. The participant signed an Informed Consent Form approved by the Ethics Research Committee of the Faculty of Ceilândia of the University of Brasília, protocol number 4.133.829, CAAE 30735320.4.0000.8093.

At the beginning and end of the treatment, the participant completed the following tests: Mini-Mental State Examination (MMSE; Brazilian version)^(11)^; free-recall subtest from the Montreal Communication Assessment Battery (MCAB)^(12)^; repetition subtest from the Boston Diagnostic Aphasia Examination (BDAE)^(13)^, and the oral naming, written naming, oral comprehension, and written comprehension subtests from the Montreal-Toulouse Language Assessment Battery (MTL)^(14)^. The standard tests were performed at three stages: (1) before the traditional treatment; (2) immediately after the traditional treatment (without tDCS); (3) 5 months after the end of the tDCS treatment. Table 1 presents the participant’s performance in the standard tests. The participant presented phonological and semantic errors both in the oral language and in the writing, in addition to speech manifestations of features of conduction aphasia, like anomies, semantic and phonemic paraphasias, paraphrases, rare neologisms, and more errors in oral repetition than in spontaneous speech. Additionally, in the writing skill tests, the participant presented anomies and plenty of literal and graphemic paragraphias, manifestations observed in deep agraphia cases.

**Table 1:** Data of assessment with standard tests before and after intervention

Standard Tests	Pre-Treatments			Post-Traditional Treatment (without TDCS)			5 Months After TDCS Treatment	
	Performance	Manifestations		Performance	Manifestations		Performance	Manifestations
								
MMSE	14/30			N/D			18/30	
MCAB								
Free-recall	0			N/D			19	
BDAE								
Repetition	4/10	Phonemic paraphasias		710	Phonemic paraphasias.		7/10	
MTL								
Oral naming (total)	16/30	Anomies, paraphasias semantic and phonemic, neologisms, paraphrases		22/30	Anomies, paraphasias semantic and phonemic, neologisms, paraphrases		17/30	Anomies, paraphrases, semantic and phonemic paraphasias
Written naming								
Nouns	2/24	Anomies, literal and graphemic paragraphia, handwriting deficits		10/24	Literal and graphemic paragraphias, paraphrases and handwriting deficits		2/24	Anomies, literal and graphemic paragraphia and handwriting deficits
Verbs	1/6			1/6			1/6	
Oral comprehension								
Words	5/5	Phonological e semantic errors.		5/5	Semantic, visual, and phonological errors.		5/5	
Sentences	8/14			10/14			8/14	
Total	13/19			15/19			13/19	
Written comprehension								
Words	2/5			3/5			3/5	
Sentences	3/8			4/8			4/8	

**Caption:** MMSE = Mini-Mental State Examination (Brucki et al.^(11)^); MCAB = Montreal Communication Assessment Battery (Fonseca et al.^(12)^); BDAE = Boston diagnostic aphasia examination (Goodglass and Kaplan^(13)^); MTL = Montreal-Toulouse Language Assessment Battery (Parente et al.^(14)^); N/D = not available

In all stages of the speech therapy monitoring, the participant demonstrated to be independent in performing basic daily life activities. As for the instrumental routine activities, he went from total to partial dependency to use the phone after the first traditional treatment period. He was able to move independently outside his home and was able to prepare his own meals. He needed assistance for housework, handling medication and money, as well as shopping. After treatment, we observed a decrease in the participant's dependence to perform these activities.

In addition to the aforementioned daily life activities, he participated passively (due to the aphasia) in regular meetings to share experiences about alcohol use disorder. He reported having participated actively in those meetings and found clear and gradual improvement when sharing his experiences with the group during and after the end of the speech therapy sessions.

The speech therapy for written language associated with tDCS consisted of five sessions of 50 min each, with two sessions every two weeks and three intensive sessions on three days on the same week (Monday, Wednesday, and Friday). The sessions occurred at the participant’s home. A set of items was applied during the intervention sessions. Each session addressed a target grapheme. Target graphemes were identified both at the assessment sessions and in the traditional treatment, representing graphemes in which oral and writing emissions by the participant were impaired. The following graphemes were addressed: <n>, <v>, <g>, <b>, and <s>. The writing intervention consisted of the computerized visual display of 30 stimuli, including 10 containing the target graphemes corresponding to each session: 10 pseudowords (5 trained and assessed; 5 trained and not assessed), 10 low-frequency regular words (5 trained and assessed; 5 trained and not assessed), and 10 irregulars (5 trained and assessed; 5 trained and not assessed). The words were selected according to their graphophonemic similarity considering that all words were disyllables, and each group contained the same number of words initiated in the target graphemes.

**Figure 1:**
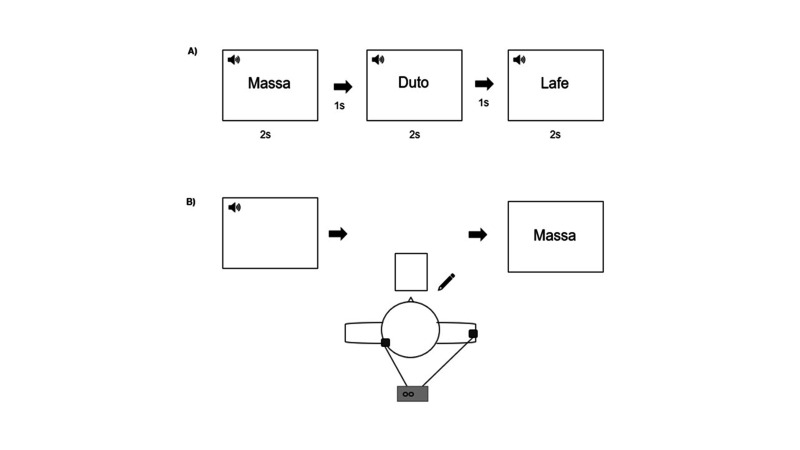
Speech and language therapy set-up (training on writing under dictation associated with transcranial direct current stimulation). (A) Visual presentation of the stimuli at the beginning of each session, and (B) view from above showing participant's position during the dictation of 30 stimuli with concurrent transcranial direct current stimulation. Dark dots on head and shoulder indicate electrode positions.

Initially, each word appeared on the computer screen for 2 s, with an interval of 1 s between the stimuli (Figure 1A). The experimenter enunciated each word concurrently with the introduction of each stimulus on the screen. After the initial presentation of the stimuli, the dictation started. The experimenter enunciated each word in the trained list three times. With pencil and eraser, the participant was encouraged to write what he heard and instructed to write as he preferred. He chose to write in block letters. After writing each stimulus, he was introduced to the correct spelling image of the respective word in block letters and encouraged to observe and compare with his elaboration (Figure 1B).

**Figure 2:**
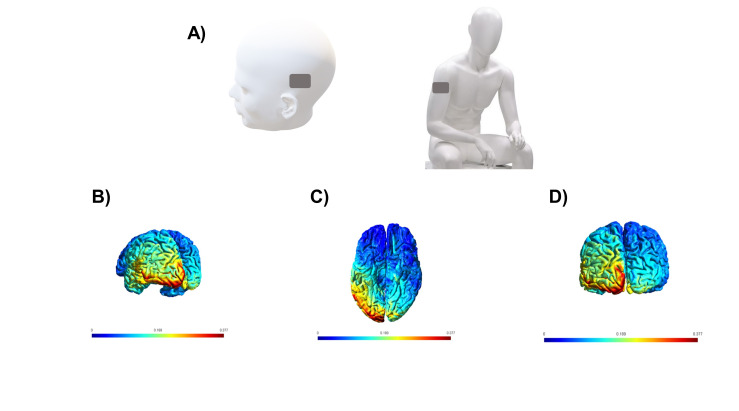
Electrode positioning during training on writing under dictation associated with tDCS (transcranial direct current stimulation). (A) The anode (positive electrode) was placed on T5 (occipitotemporal region in the 10/20 system of the electroencephalogram). The cathode (negative electrode) was placed over the right deltoid muscle. (B-D) SimNIBS simulation of the electric field induced by tDCS on cortical regions). (B) Lateral view. (C) Inferior view. (D) Posterior view. Minimum (blue) and maximum (red) electric fields, in V/m, were generated with a current of 2 mA and 35-cm2 electrodes placed over T5 and the right deltoid muscle. The simulation indicates current concentration on the left ventral occipitotemporal córtex.

In all five sessions, the tDCS application coincided with the start of the dictation task for the 30 intervention stimuli. We used electrodes of 35 cm^2^ soaked in saline solution. The anode (positive) was positioned on the T5 (International System 10/20 of encephalogram), scalp area located on the left occipitotemporal cortex. The cathode (negative) was positioned on the contralateral deltoid muscle (Figure 2A), and the stimulation with 2 mA lasted 20 min. Figure 2 also illustrates a simulation with SimNIBS indicating the range of the electric current in the skull. Each session had a total duration of 50 min. TDCS device assembly and general instructions to the participant took 10 min. A professional physiotherapist applied the tDCS for 20 min simultaneously to the behavioral treatment through dictation, which, in turn, was applied by a speech therapist. TDCS was completed 20 min later, and the dictation training continued until the participant finished the writing of the 30 stimuli, at an average duration of 40 min.

After the first TDCS session, the participant reported a mild headache, which did not occur after the following sessions. Such symptoms can be related both to the tDCS^(6)^ application and the visual effort of the writing activity, which occur during an only session in the traditional treatment, later constituting most of the tDCS treatment session. It is worth pointing out that the participant wore glasses adapted to his visual impairment.

For the treatment effects to be monitored, the participant completed an assessment dictation composed of 40 stimuli, including 10 pseudowords (5 trained and 5 untrained), 10 low-frequency regular words (5 trained and 5 untrained), and 20 irregular words (10 trained and 10 untrained). Untrained words are stimuli present in the assessment dictation that were not used at the training sessions. Untrained words were included in order to assess the potential generalization of treatment results. The main variable of interest was the participant’s ability to write the 40 items in the assessment dictation. The application of the assessment dictation occurred at three stages: (1) before the tDCS treatment, (2) immediately after the intensive treatment, and (3) 5 months after the end of the treatment (Table 2).

**Table 2:** Performance in the dictation before tDCS intervention immediately after intensive training and 5 months after the end of the training

Type of Stimulus	Pre-Intervention	Post-Intervention	5 Months After Intervention
Pseudowords			
Trained	1/5 (20%)	3/5 (60%)	3/5 (60%)
Untrained	0/5 (0%)	3/5 (60%)	2/5 (20%)
Regular Words (Low Frequency)			
Trained	0/5 (0%)	4/5 (80%)	1/5 (20%)
Untrained	1/5 (20%)	1/5 (20%)	1/5 (20%)
Irregular Words			
Trained	2/10 (20%)	8/10 (80%)	1/10 (10%)
Untrained	2/10 (20%)	3/10 (30%)	2/10 (20%)

**Caption:** Performance measured in number and percentage of correctly written words

The results indicated improvement in the writing activity right after the end of the written language intervention associated with tDCS (Table 2). Before the treatment, the participant had correctly written 1 pseudoword (20%), 1 low-frequency regular word (20%), and 4 irregular words (20%). His low performance in pseudowords and low-frequency regular words indicates failure in the use of the phonological route. The low performance in irregular words indicates failure in the use of the phonological route^(3,4)^. The presence of deficits in both routes is characteristic of deep agraphia^(1)^. In this case, the participant showed worse performance on words that rely on the phonological route.

Immediately after the written language intervention associated with tDCS, we found a writing improvement in the three types of stimuli. The number of correctly written responses increased for pseudowords (trained: from 20% to 60%) and for low-frequency regular words (trained: from 20% to 80%), indicating an improvement in the phonological route. The number of correctly written responses also increased for irregular words (trained: from 20% to 80%), indicating an improvement in the lexical route. As for the untrained words, a significant increase occurred only for pseudowords (untrained: from 0% to 60%), suggesting treatment generalization for stimuli that depend especially on the phonological route.

The assessment performed 5 months after the written language treatment associated with tDCS indicated partial maintenance of the initial improvement; however, only for the phonological route. The participant correctly produced 5 pseudowords relative to 1 pseudoword before the intervention and 2 low-frequency regular words relative to 1 low-frequency regular word before intervention. However, for irregular words, the better performance observed right after the intervention was not maintained (3 correct responses 5 months after the intervention compared to 4 correct responses before the intervention).

In the standard tests, the participant’s performance improved for some skills. In the MMSE^(11)^, the participant’s performance before the treatments (traditional and TDCS) was below expected: 14 points, below the average and the cutoff score according to Brazilian studies^(11,15)^. Five months after the end of the tDCS intervention, the MMSE had an increase of 4 points, reaching 18 points, a score corresponding to the cutoff score in the normative Brazilian study^(15)^. In particular, the performance in the drawing copy item was considered normal in the initial assessment, thus suggesting that the participant did not present severe deficits in visual and motor processing before both the traditional treatment and the tDCS.

The free-recall task from the MCAB Battery^(12)^ pointed to significant improvement. Before the traditional speech therapy, the participant could not recall any word, thus indicating lexical access failure (cutoff score: 15). However, in the assessment performed 5 months after the end of the tDCS intervention, the participant recalled 19 words, a performance regarded as normal considering the participant’s age and schooling. Neither the MMSE^(11)^ nor the free-recall subtest from the MCAB Battery^(12)^ were applied immediately after the end of the traditional treatment.

In the repetition subtest from the BDAE^(13)^, the participant’s performance was below the expected in the initial assessment (cutoff score: 10), suggesting a condition of conduction aphasia, with marked alteration in oral repetition and mild alteration in oral emission and comprehension (MTL)^(14)^. As for oral communication, an improvement was indicated in the assessment performed after the traditional treatment, which persisted over the 5 months following the end of the TDCS training; however, the score remained below normal.

In the MTL subtests^(14)^, the participant’s performance was below normal in the oral naming task before the beginning of the treatment (cutoff score: 26,43). His performance improved after the end of the traditional treatment. Despite the drop in performance indicated in the assessment carried out 5 months after the end of the tDCS intervention, performance was still better than baseline. In the written naming task, despite the significant improvement right after the traditional treatment concerning the initial assessment, the participant’s performance assessed 5 months after the end of the tDCS intervention reverted to the same score obtained on the baseline. Despite the improvement indicated in all assessments, the score remained below normal (cutoff score: 16,79). In the written naming task, performance for verbs also remained below normal, and did not improve on any of the three assessments performed (cutoff score: 4,15).

In the subtest of oral comprehension of words from the MTL battery^(14)^, the participant’s performance in the initial assessment was normal, based on his age and schooling (cutoff score: 4,37). A mild improvement occurred after the traditional treatment, followed by a drop in the initial score 5 months after the end of the tDCS intervention. In contrast, in the subtest of oral comprehension of sentences, his performance in the initial assessment was below normal (cutoff score: 10,11). After the traditional treatment, the performance improvement was normal, but another drop occurred in the assessment carried out 5 months after the tDCS treatment, returning to baseline – below normal. In the subtests of written comprehension of words and sentences from the MTL battery^(14)^, the participant’s performance remained below normal in the initial assessment (cutoff score for writing comprehension of words: 4,10; the cutoff score for writing comprehension of sentences: 6.04). The traditional treatment was followed by improved performance in both of the subtests, but still below normal. The improvement persisted 5 months after the end of the treatment.

Regarding his self-perception, the participant reported improvements in his writing skills. After the treatment, he was able to make use of a shopping list and was able to write brief messages about everyday activities for his immediate family. In addition, he observed an overall improvement in oral communication.

## DISCUSSION

In this study, we report the clinical improvement, in a writing under dictation task, of an elderly individual with conduction aphasia and deep agraphia. The patient underwent a speech therapy for written language associated with tDCS. The trained stimuli were chosen in order to stimulate the phonological and lexical routes. The results suggest improvement in both routes, although the effect on the phonological route was more evident and persisted for 5 months following the treatment. The improvement in writing was relevant when considering the participant’s chronic deficit in language skills of emission and comprehension in the standard tests (e.g., MTL).

The position of the positive electrode (in T5) was guided by computer simulation (SimNIBS) to maximize the electric potential on the left occipitotemporal cortex. Recent models of written language processing point to an important role of the left occipitotemporal cortex in the phoneme-grapheme conversion and lexical processing^(4)^. By stimulating this area, we aimed to maximize the training of phoneme-grapheme conversion and lexical access.

Processing via the phonological route benefited the most from the treatment, according to the response during the dictation of pseudowords and low-frequency regular words. Such improvement in dictation writing may have resulted from the stronger engagement of the occipitotemporal cortex under tDCS during the phoneme-grapheme conversion, which is required for the correct writing of the target word via phonological route. As for irregular words, though, it is necessary to access the lexical route, since prior knowledge of spelling is required, and basing the decision only on the phonological route can lead to writing errors. Furthermore, despite the drop in performance for irregular words 5 months following the intervention, the results indicate long-term processing improvement through the phonological route. TDCS studies in individuals with aphasia indicate that cumulative application of stimulation can be required to achieve long-term effects^(7)^.

Our study encompassed only five sessions, which is enough to achieve a late benefit. The transitory nature of the effect indicated in some of the tests (reversion to the baseline 5 months after the end of the treatment) can be related to the number of sessions. 

Current evidence shows that individuals with chronic conditions after stroke tolerate well the use of this instrument, demonstrating therapeutic benefits in aphasia cases^(6,7)^. Regarding the generalization of the intervention effects, a significant improvement was found only for the untrained stimuli that depend on the phonological route.

The lesser benefit in the lexical route and the return to baseline scores in certain tests after a long post-intervention period may be explained by the participant’s schooling level and prior reading habits, since the use of the lexical route requires regular use of spelling.

The study has some limitations regarding the initial assessment of the participant due to the absence of detailed analyses on the input and output skills of oral language and handwriting, which could provide further information on the benefits of TDCS on oral and written communication. The potential separate effects of each intervention were not assessed as well (i.e., only writing training vs. only neuromodulation). Pre- and post-assessment of the tDCS intervention, without concomitant writing training, would allow us to establish tDCS’ separate effect on the participant’s performance more precisely. Furthermore, the standard tests were not performed immediately after the tDCS intervention, which prevented us from comparing the patient’s performance in standard tests before and after the neuromodulation intervention. This study encompassed two sessions every two weeks and three intensive sessions in three days of the same week, and the interval between the sessions may have influenced the results. Greater proximity between sessions, especially the initial ones, could have enhanced the participant's performance during the applied tests. The participant’s initial complaint, his improvement during the tests, and his self-perception of improvement all suggest a clinical and functional improvement in the patient’s writing skills. Nonetheless, we highlight that such results should be interpreted with caution considering the small number of items assessed. Applying an assessment with a larger number of items could have provided greater response variability.

## FINAL REMARKS

A treatment involving writing under dictation associated with tDCS on the left occipitotemporal cortex in an individual with deep agraphia provided a marked improvement in the clinical assessment of writing emission of pseudowords and a small improvement in the writing of low-frequency regular words, both stimuli processed by the phonological route. The stimulation provided an immediate benefit to the writing of irregular words accessed via the lexical route. The participant also presented a small improvement for untrained stimuli, thus suggesting generalization. However, the follow-up assessment carried out 5 months later indicated that the gains observed right after the training remained only for words associated with the phonological route. Considering the severity and chronicity of the case, the results are promising and suggest that tDCS associated with writing treatment represents a possible clinical alternative for patients with deep agraphia.

## References

[1] Carthery MT, Parente MAMP, Ortiz KZ (2010). Distúrbios neurológicos adquiridos..

[2] Carthery MT, Parente MAMP, Ortiz KZ (2010). Distúrbios neurológicos adquiridos..

[3] Rapcsak SZ, Henry ML, Teague SL, Carnahan SD, Beeson PM (2007). Do dual-route models accurately predict reading and spelling performance in individuals with acquired alexia and agraphia?. Neuropsychologia.

[4] Rapcsak SZ, Beeson PM, Hillis AE (2015). The handbook of adult language disorders..

[5] Ripamonti E, Frustaci M, Zonca G, Aggujaro S, Molteni F, Luzzatti C (2018). Disentangling phonological and articulatory processing: a neuroanatomical study in aphasia. Neuropsychologia.

[6] Lefaucheur JP, Antal A, Ayache SS, Benninger DH, Brunelin J, Cogiamanian F (2017). Evidence-based guidelines on the therapeutic use of transcranial direct current stimulation (tDCS). Clin Neurophysiol.

[7] Holland R, Crinion J (2012). Can tDCS enhance treatment of aphasia after stroke?. Aphasiology.

[8] Nitsche MA, Paulus W (2000). Excitability changes induced in the human motor cortex by weak transcranial direct current stimulation. J Physiol.

[9] Broeder S, Nackaerts E, Cuypers K, Meesen R, Verheyden G, Nieuwboer A (2019). tDCS-Enhanced Consolidation of writing skills and its associations with cortical excitability in parkinson disease: a pilot study. Neurorehabil Neural Repair.

[10] Fenner AS, Webster KT, Ficek BN, Frangakis CE, Tsapkini K (2019). Written Verb Naming Improves After tDCS Over the Left IFG in Primary Progressive Aphasia. Front Psychol.

[11] Brucki SMD, Nitrini R, Caramelli P, Bertolucci PHF, Okamoto IH (2003). Sugestões para o uso do mini-exame do estado mental no Brasil. Arq Neuropsiquiatr.

[12] Fonseca RP, Parente MAMP, Côté H, Joanette Y (2007). Processo de adaptação da bateria Montreal de avaliação da comunicação: bateria MAC ao português brasileiro. Psicol Reflex Crit.

[13] Goodglass H, Kaplan EF (1984). The assessment of aphasia and related disorders..

[14] Parente MAMP, Ortiz KZ, Soares ECS, Scherer L, Fonseca R, Joanette Y (2016). Bateria Montreal-Toulouse de Avaliação da Linguagem-Bateria MTL-Brasil..

[15] Bertolucci PH, Brucki S, Campacci SR, Juliano Y (1994). O mini-exame do estado mental em uma população geral: impacto da escolaridade. Arq Neuropsiquiatr.

