# Supplementary material for: Effects of Transcranial Direct Current Stimulation in writing: a case report of deep agraphia
Source: Codas. 2022 Feb 2;34(4):e20200319. doi: 10.1590/2317-1782/20212020319 (PMC9886107; doi:10.1590/2317-1782/20212020319)
Supplement: Translation

# Translation: Efeitos da Estimulação Transcraniana por Corrente Contínua na escrita: um relato de caso de agrafia profunda

**Authors:** Nathani Cristine do Carmo Ramos, Cláudia Aparecida Pietrobon, Ricardo Marcio Garcia Rocha, Luciana Lilian Louzada Martini, Luciano Grüdtner Buratto, Maysa Luchesi Cera

**Affiliations:** 1 Programa de Pós-Graduação em Ciências do Comportamento, Universidade de Brasília – UnB – Brasília (DF), Brasil.; 2 Secretaria de Saúde do Distrito Federal – SES/DF – Brasília (DF), Brasil.; 3 Faculdade de Ceilândia, Universidade de Brasília – UnB – Brasília (DF), Brasil.

**Keywords:** Agrafia, Acidente Vascular Cerebral, Escrita manual, Estimulação Transcraniana por Corrente Contínua, Afasia

## Abstract

Apresentamos o relato de caso de um participante do sexo masculino, 61 anos, com afasia de condução crônica e agrafia profunda após acidente vascular cerebral isquêmico que recebeu treinamento de escrita sob ditado associado à estimulação transcraniana por corrente contínua. O tratamento consistiu em cinco sessões de 50 minutos de escrita sob ditado com aplicação de 2 mA de estimulação transcraniana por corrente contínua anódica durante 20 minutos sobre o córtex occipitotemporal esquerdo. O participante apresentou melhora na produção escrita de pseudopalavras e de palavras regulares de baixa frequência, via rota fonológica, além de uma pequena melhora na produção de palavras irregulares, via rota lexical. Após o treinamento, houve também pequena melhora da escrita de estímulos não treinados, sugerindo generalização. Na avaliação realizada 5 meses após o término do tratamento, o benefício foi mantido para estímulos processados via rota fonológica. Os resultados são promissores dada a gravidade e cronicidade do caso e sugerem que a estimulação transcraniana por corrente contínua associada à terapia de escrita representa possível alternativa clínica para pacientes com agrafia profunda.

## INTRODUÇÃO

Agrafia é um transtorno adquirido de escrita caracterizado por alterações na escrita espontânea e sob ditado, que não pode ser atribuído somente a déficits perceptuais ou motores^(1)^. O diagnóstico é baseado em uma avaliação de quais processos envolvidos na linguagem escrita estão alterados ou preservados^(2)^.

Além da aplicação de testes de escrita espontânea, nomeação escrita, escrita sob ditado e cópia^(2)^, essa avaliação deve ser constituída por variados graus de demanda sobre representações fonológicas (e.g., sequência de grafemas da palavra) e lexicais (e.g., forma da palavra). Pseudopalavras, por exemplo, requerem processamento fonológico preservado – via rota fonológica – para que possam ser escritas corretamente^(3)^. Em contraste, palavras irregulares exigem acesso a representações ortográficas – via rota lexical –, pois apenas seguir regras fonológicas pode levar a erros na escrita^(3,4)^.

Indivíduos com agrafia lexical apresentam alteração na escrita de palavras irregulares e cometem erros de regularização pela utilização do procedimento de conversão fonografêmica^(1,2)^. O prejuízo na escrita de palavras de baixa frequência ou de pseudopalavras são características da agrafia fonológica, enquanto a agrafia profunda é caracterizada pela ocorrência de erros em abundância no uso da rota lexical e pela incapacidade de utilizar a rota fonológica^(1,2)^.

Em termos neurais, as evidências apontam para uma divisão de tarefas entre diferentes regiões corticais para execução da escrita. O processamento fonológico está associado a uma rede perisilviana esquerda que inclui o giro temporal superior, a parte posterior do giro supramarginal e o giro de Heschl^(5)^. O processamento lexical, por outro lado, está associado à atividade em regiões occipitotemporais^(4)^. Lesões focais em regiões responsáveis pelas rotas fonológica e lexical podem ocasionar déficits específicos de escrita. Por exemplo, lesões no córtex occipitotemporal esquerdo resultam em prejuízo desproporcional na escrita de palavras irregulares (rota lexical) em comparação com palavras regulares (rotas lexical e fonológica) ou pseudopalavras (rota fonológica)^(1)^. A reabilitação, nestes casos, pode ser dirigida à melhora do sistema semântico e lexical ou à melhora da rota de conversão fonografêmica, ou ambas, a fim de reorganizar a atividade de escrita^(2)^.

Na última década, pesquisadores têm avaliado a associação de tratamentos tradicionais para favorecer a comunicação com técnicas de neuromodulação na tentativa de potencializar seus efeitos clínicos^(6)^. Em pessoas com afasia de Broca após acidente vascular cerebral, por exemplo, o uso concomitante de estimulação transcraniana por corrente contínua (ETCC) com terapias de nomeação têm resultado em melhorias no desempenho em tarefas de nomeação^(6,7)^.

A ETCC é uma técnica de estimulação não invasiva que envolve a aplicação de uma corrente elétrica direta fraca (1–2 mA) por meio de dois ou mais eletrodos colocados sobre o couro cabeludo. O campo elétrico constante resultante da estimulação influencia a atividade elétrica do tecido neural subjacente^(8)^. Em geral, a ETCC anódica (eletrodo positivo) tem efeito excitatório, e a ETCC catódica (eletrodo negativo) tem efeito inibitório sobre as regiões corticais afetadas^(6)^.

A maior parte dos estudos com ETCC até o momento avaliou o desempenho de pessoas com prejuízos em linguagem oral por meio de estimulação de áreas tradicionalmente associadas à fala, como a área de Broca^(6,7)^. Estudos com ETCC em indivíduos com alterações na escrita ainda são escassos. Nestes estudos, os alvos da estimulação foram áreas associadas aos aspectos motores da escrita (córtex motor primário esquerdo em pessoas com doença de Parkinson)^(9)^ ou áreas de convergência entre fala e escrita (giro frontal inferior esquerdo em indivíduos com afasia progressiva primária)^(10)^. Em nenhum desses estudos, estimularam-se áreas posteriores, como o córtex occipitotemporal esquerdo, associado ao processo de conversão fonema-grafema e ao acesso lexical em modelos cognitivos neuropsicológicos de escrita^(4)^.

O objetivo do presente estudo é apresentar o relato de caso de um idoso com agrafia profunda que participou de um treinamento de escrita sob ditado associado à ETCC aplicada sobre o córtex occipitotemporal esquerdo. A hipótese desta intervenção é que a ETCC anódica em região occipitotemporal esquerda será benéfica no treinamento de palavras que requeiram o processamento das rotas fonológica e lexical.

## APRESENTAÇÃO DO CASO CLÍNICO

O homem de 61 anos, 5 anos de estudo formal, cozinheiro, ex-etilista, ex-tabagista, com diabetes mellitus tipo 2 e hipertensão arterial controladas, insuficiência renal crônica e deficiência visual corrigida, apresentou histórico de transtornos da comunicação, afasia de condução e agrafia profunda após acidente vascular cerebral isquêmico (AVCI), em região posterior dos lobos parietal e temporal à esquerda.

Após 6 anos da data de ocorrência do AVCI, o indivíduo iniciou a reabilitação fonoaudiológica tradicional semanal. Durante o tratamento inicial, habilidades de emissão e compreensão oral e escrita foram estimuladas. Após 6 meses de tratamento tradicional semanal, o participante apresentou melhora funcional significativa de emissão oral. Nesse período, o participante deixou de aderir ao tratamento, pois relatou estar satisfeito com os ganhos em sua comunicação oral, que era seu principal objetivo. No entanto, sua escrita continuava prejudicada. Com o intuito de promover melhora no desempenho da linguagem escrita, foi proposto ao participante o tratamento intensivo de escrita associado à ETCC. O participante assinou o Termo de Consentimento Livre e Esclarecido para participar deste estudo, que foi aprovado pelo Comitê de Ética em Pesquisa da Faculdade de Ceilândia da Universidade de Brasília, sob parecer número 4.133.829, CAAE 30735320.4.0000.8093.

No início e ao final do tratamento, o participante foi submetido aos seguintes testes: Mini Exame do Estado Mental (MEEM; versão brasileira)^(11)^; subteste de evocação livre da Bateria Montreal de Avaliação da Comunicação (MAC)^(12)^; subteste de repetição do Teste de Boston para Diagnóstico de Afasias (BDAE)^(13)^ e subtestes de nomeação e compreensão oral e escrita da Bateria Montreal-Toulouse de Avaliação da Linguagem (MTL)^(14)^. Os testes padronizados foram realizados em três momentos: (1) antes do tratamento tradicional; (2) logo após o tratamento tradicional (sem ETCC); (3) 5 meses após o término do tratamento associado à ETCC. A Tabela 1 apresenta o desempenho do participante nestes testes padronizados. O participante apresentou erros fonológicos e semânticos tanto na linguagem oral quanto na escrita. O participante também apresentou manifestações na fala características de afasia de condução como, por exemplo, anomias, parafasias semânticas e fonêmicas, paráfrases, raros neologismos e mais erros na repetição oral em comparação com a fala espontânea. Além disso, nos testes de habilidade de escrita, o participante apresentou anomias e abundantes paragrafias literais e grafêmicas, manifestações observadas em casos de agrafia profunda.

**Tabela 1:** Dados de avaliação com testes padronizados antes e após a intervenção

Testes Padronizados	Antes dos Tratamentos			Após Tratamento Tradicional (sem ETCC)			5 Meses Após Tratamento com ETCC	
	Desempenho	Manifestações		Desempenho	Manifestações		Desempenho	Manifestações
								
MEEM	14/30			N/D			18/30	
MAC								
Evocação Livre	0			N/D			19	
BDAE								
Repetição	4/10	Parafasias fonêmicas		7/10	Parafasias fonêmicas.		7/10	
MTL								
Nomeação oral (total)	16/30	Anomias, parafasias semânticas e fonêmicas, neologismos, paráfrases		22/30	Anomias, parafasias semânticas e fonêmicas, neologismos, paráfrases		17/30	Anomias, paráfrases, parafasias semânticas e fonêmicas
Nomeação escrita								
Substantivos	2/24	Alterações de traçado, anomias, paragrafias literais e grafêmicas		10/24	Alterações de traçado, paragrafias literais, grafêmicas e paráfrases		2/24	Alterações de traçado, anomias, paragrafias literais e grafêmicas
Verbos	1/6			1/6			1/6	
Compreensão oral								
Palavras	5/5	Apresentou erros fonológicos e erro semântico.		5/5	Apresentou erros semânticos, visuais e fonológicos.		5/5	
Frases	8/14			10/14			8/14	
Total	13/19			15/19			13/19	
Compreensão escrita								
Palavras	2/5			3/5			3/5	
Frases	3/8			4/8			4/8	
			

Legenda: MEEM = Mini Exame do Estado Mental (Brucki et al.^(11)^); MAC = Bateria Montreal de Avaliação da Comunicação (Fonseca et al.^(12)^); BDAE = Teste de Boston para Diagnóstico de Afasias (Goodglass e Kaplan^(13)^); MTL = Bateria Montreal-Toulouse de Avaliação da Linguagem (Parente et al.^(14)^); N/D = não disponível

Em todos os momentos de acompanhamento fonoaudiológico, o participante demonstrou independência para as atividades básicas de vida diária. Quanto às atividades instrumentais de vida diária, passou de dependência total para parcial para utilizar o telefone após o primeiro período de terapia tradicional. Apresentava independência para locomoção fora de casa e para preparar a própria refeição. Necessitava de assistência para realização de trabalho doméstico, manipulação da medicação e do dinheiro e para fazer compras. Após a terapia, observou-se diminuição da dependência do participante para a execução dessas atividades.

Além das atividades de vida diária descritas anteriormente, participava, passivamente (devido à afasia) de reuniões regulares para compartilhamento de experiências sobre o transtorno do uso de álcool. Relatou participação ativa e melhora evidente e gradativa nas conversas deste grupo durante e após o processo fonoaudiológico completo.

A terapia fonoaudiológica de linguagem escrita associada à ETCC consistiu em cinco sessões de 50 min, sendo duas sessões quinzenais e três sessões intensivas em três dias da mesma semana (segunda, quarta e sexta-feira). As sessões foram realizadas na casa do participante. Um conjunto de itens foi aplicado durante as sessões de intervenção. Em cada sessão, trabalhou-se um grafema-alvo. Grafemas-alvo foram identificados nas sessões de avaliação e de terapia tradicional e representam grafemas cuja emissão oral e escrita pelo participante estavam prejudicadas. Os grafemas trabalhados foram <n>, <v>, <g>, <b> e <s>. A intervenção escrita consistiu na apresentação visual computadorizada de 30 estímulos, dentre eles 10 eram estímulos que continham o grafema alvo correspondente a cada sessão: 10 pseudopalavras (5 treinadas e avaliadas; 5 treinadas e não avaliadas), 10 palavras regulares de baixa frequência (5 treinadas e avaliadas; 5 treinadas e não avaliadas) e 10 irregulares (5 treinadas e avaliadas; 5 treinadas e não avaliadas). As palavras foram selecionadas conforme semelhança grafofonêmica, todas as palavras eram dissílabas e cada grupo continha a mesma quantidade de palavras iniciadas pelos grafemas-alvo.

Inicialmente, cada palavra apareceu na tela do computador por 2 s, com intervalo de 1 s entre os estímulos (Figura 1A). O experimentador enunciou cada palavra de maneira concomitante à apresentação de cada estímulo na tela. Após esta apresentação inicial dos estímulos, o ditado foi iniciado. O experimentador enunciou três vezes cada palavra da lista treinada. O participante, munido de lápis e borracha, foi encorajado a escrever o que ouviu. Ele foi instruído a escrever do modo que preferisse. O participante escolheu escrever em letra de forma. Após a escrita de cada estímulo, foi apresentada ao sujeito a imagem da escrita correta da respectiva palavra em letra de forma e ele foi incentivado a observar e comparar com a sua elaboração (Figura 1B).

**Figura 1:** Demonstração da intervenção fonoaudiológica. (A): Apresentação inicial dos estímulos e (B) vista superior da posição do participante durante o ditado composto por 30 estímulos com aplicação simultânea de estimulação transcraniana por corrente contínua. Os pontos escuros indicam a posição dos eletrodos.

Nas cinco sessões, a aplicação da ETCC coincidiu com o início da tarefa de ditado dos 30 estímulos da intervenção. Foram utilizados eletrodos de 35 cm^2^ embebidos em solução salina. O ânodo (positivo) foi posicionado em T5 (Sistema Internacional 10/20 do encefalograma), região do escalpo localizada sobre o córtex occipitotemporal esquerdo. O cátodo (negativo) foi posicionado sobre o músculo deltoide contralateral (Figura 2A) e a estimulação com 2 mA foi fornecida durante 20 min. A Figura 2 também ilustra uma simulação com o software SimNIBS indicando o alcance da corrente elétrica no crânio. Cada sessão teve duração total de 50 min. Foram necessários 10 min para a montagem do aparelho de ETCC e para fornecer instruções gerais ao participante. A ETCC foi aplicada durante 20 min por um profissional fisioterapeuta simultaneamente ao tratamento comportamental, por meio do ditado, aplicado por uma fonoaudióloga. A ETCC foi finalizada após 20 min enquanto o treino com o ditado ocorreu até que o participante completasse a escrita dos 30 estímulos, com duração média de 40 min.

**Figura 2:** Posicionamento dos eletrodos utilizados no tratamento de ditado associado à ETCC. (A) O ânodo (eletrodo positivo) foi posicionado em T5 (região occipitotemporal no sistema 10/20 do eletroencefalograma). O cátodo (eletrodo negativo) foi posicionado sobre o músculo deltoide direito. (B-D) Simulação realizada no software SimNIBS. (B) Vista lateral. (C) Vista inferior. (D) Vista posterior. O campo elétrico mínimo (azul) e máximo (vermelho), em V/m, foi gerado com corrente de 2 mA e eletrodos de 35 cm^2^ sobre T5. A simulação indica concentração de corrente no córtex occipitotemporal ventral esquerdo.

Após a primeira sessão com ETCC o participante relatou leve cefaleia, o que não ocorreu nas demais sessões. Este sintoma pode ter relação tanto com a aplicação da ETCC^(6)^ quanto com o esforço visual em atividade de escrita, que no tratamento tradicional foi desenvolvida apenas durante um período da sessão e passou a compor a maior parte da sessão no tratamento com ETCC. É importante ressaltar que o participante usou óculos adaptados à sua deficiência visual.

Para acompanhamento dos efeitos do tratamento, o participante passou por um ditado de avaliação composto por 40 estímulos, sendo 10 pseudopalavras (5 treinadas e 5 não treinadas), 10 palavras regulares de baixa frequência (5 treinadas e 5 não treinadas) e 20 palavras irregulares (10 treinadas e 10 não treinadas). Palavras não treinadas são estímulos presentes no ditado de avaliação, mas que não foram utilizados nas sessões de treinamento, com o intuito de verificar o potencial de generalização dos resultados do tratamento. A principal variável de interesse para a análise de dados deste estudo foi a capacidade do participante de escrever os 40 itens que compõem este ditado de avaliação. A aplicação deste ditado de avaliação foi realizada em três momentos: (1) antes do tratamento com ETCC; (2) logo após o tratamento intensivo; (3) 5 meses após o término do tratamento (Tabela 2).

**Tabela 2:** Desempenho no ditado antes da intervenção com ETCC, logo após o treino intensivo e 5 meses após o término do treino

Tipo de Estímulo	Antes da Intervenção	Após a Intervenção	5 Meses Após a Intervenção
Pseudopalavras			
Treinadas	1/5 (20%)	3/5 (60%)	3/5 (60%)
Não Treinadas	0/5 (0%)	3/5 (60%)	2/5 (20%)
Palavras Regulares (Baixa Frequência)			
Treinadas	0/5 (0%)	4/5 (80%)	1/5 (20%)
Não Treinadas	1/5 (20%)	1/5 (20%)	1/5 (20%)
Palavras Irregulares			
Treinadas	2/10 (20%)	8/10 (80%)	1/10 (10%)
Não Treinadas	2/10 (20%)	3/10 (30%)	2/10 (20%)

**Legenda:** Desempenho medido em número e porcentagem de acertos.

Os resultados indicaram melhora na atividade de escrita logo após o término da intervenção de linguagem escrita associada à ETCC (Tabela 2). Antes do tratamento, o participante acertou 1 pseudopalavra (20%), 1 palavra regular de baixa frequência (20%) e 4 palavras irregulares (20%). O baixo desempenho em pseudopalavras e palavras regulares de baixa frequência indica falha no uso da rota fonológica, e o baixo desempenho em palavras irregulares indica falha no uso da rota lexical^(3,4)^. A presença de déficits nas duas rotas é característica de agrafia profunda^(1)^. No presente caso, houve pior desempenho da rota fonológica.

Logo após a intervenção de linguagem escrita associada à ETCC, houve melhora na escrita dos três tipos de estímulos. O número de acertos aumentou para pseudopalavras (treinadas: de 20% para 60%) e palavras regulares de baixa frequência (treinadas: de 20% para 80%), indicando melhora na rota fonológica. O número de acertos também aumentou para palavras irregulares (treinadas: de 20% para 80%), indicando melhora na rota lexical. Para palavras não treinadas, houve aumento considerável apenas para pseudopalavras (não treinadas: de 0% para 60%), sugerindo generalização do tratamento para estímulos que dependem principalmente da rota fonológica.

Na avaliação realizada 5 meses após a terapia de linguagem escrita associada à ETCC, observou-se manutenção parcial da melhora inicialmente observada, mas apenas na rota fonológica. O participante obteve 5 acertos em pseudopalavras, comparados a 1 acerto antes da intervenção, e 2 acertos em palavras regulares de baixa frequência, comparados a 1 acerto antes da intervenção. Para palavras irregulares, no entanto, o aumento no desempenho observado logo após a intervenção não se manteve (3 acertos 5 meses após a intervenção comparados a 4 acertos antes da intervenção).

Nos testes padronizados, o desempenho do participante melhorou em algumas habilidades. No MEEM^(11)^, o desempenho do participante antes dos tratamentos (tradicional e com ETCC) estava abaixo do esperado: 14 pontos, abaixo da mediana e do valor de corte dos estudos brasileiros^(11,15)^. Após 5 meses do término da intervenção com ETCC, observou-se aumento de 4 pontos no MEEM, totalizando 18 pontos, escore correspondente ao valor de corte do estudo brasileiro normativo^(15)^. Em particular, o desempenho no item de cópia de desenho foi considerado normal na avaliação inicial, o que sugere que o participante não apresentava déficits graves em processamento visual e motor antes do tratamento tradicional e do tratamento com ETCC.

Na tarefa de evocação livre da Bateria MAC^(12)^, houve melhora expressiva. Antes do tratamento fonoaudiológico tradicional, o participante não evocou nenhuma palavra, o que indicou falha no acesso lexical (pontuação de corte: 15). Entretanto, na avaliação realizada 5 meses após o término da intervenção com ETCC, o participante evocou 19 palavras, desempenho considerado normal de acordo com a idade e escolaridade do participante. O MEEM^(11)^ e o subteste de evocação livre da Bateria MAC^(12)^ não foram aplicados logo após o término do tratamento tradicional.

No subteste de repetição do BDAE^(13)^, o participante apresentou desempenho abaixo do esperado na avaliação inicial (pontuação de corte: 10), sugerindo um quadro de afasia de condução, com alteração marcada em repetição oral, leve alteração de emissão e compreensão oral (MTL)^(14)^. Quanto à comunicação oral, houve melhora na avaliação realizada após o tratamento tradicional, que se manteve 5 meses após o término do treinamento com ETCC. No entanto, a pontuação ainda permaneceu abaixo da normalidade.

Nos subtestes da MTL^(14)^, o participante apresentou desempenho abaixo da normalidade na tarefa de nomeação oral antes do início do tratamento (pontuação de corte: 26,43). Houve melhora no desempenho após o término do tratamento tradicional. Apesar da queda no desempenho de acordo com a avaliação realizada 5 meses após o término da intervenção com ETCC, ainda pode-se observar melhor desempenho comparado à linha de base. No subteste de nomeação escrita de substantivos, apesar de haver uma melhora expressiva logo após o tratamento tradicional comparado com a avaliação inicial, o desempenho do participante avaliado 5 meses após o término da intervenção com ETCC regrediu para a mesma pontuação obtida na linha de base. Em todas as avaliações, apesar da melhora observada, a pontuação permaneceu abaixo da normalidade (pontuação de corte: 16,79). Na tarefa de nomeação escrita de verbos, o desempenho permaneceu abaixo da normalidade e não foi observada melhora no desempenho durante as três avaliações realizadas (pontuação de corte: 4,15).

No subteste de compreensão oral de palavras da bateria MTL^(14)^, o desempenho do participante na avaliação inicial estava dentro da normalidade, de acordo com a idade e escolaridade (pontuação de corte: 4,37). Houve pequena melhora após o tratamento tradicional, seguida de queda para a pontuação inicial 5 meses após o término da intervenção com ETCC. No subteste de compreensão oral de frases, por outro lado, o desempenho na avaliação inicial estava abaixo da normalidade (pontuação de corte: 10,11). Após o tratamento tradicional, houve melhora, com desempenho dentro da normalidade, mas houve nova queda na avaliação realizada 5 meses após o tratamento com ETCC, voltando à pontuação de linha de base, abaixo da normalidade. Nos subtestes de compreensão escrita de palavras e de frases da bateria MTL^(14)^, o desempenho do participante ficou abaixo da normalidade na avaliação inicial (pontuação de corte para compreensão escrita de palavras: 4,10; pontuação de corte para compreensão escrita de frases: 6,04). Após o tratamento tradicional, observou-se melhora no desempenho nos dois subtestes, porém ainda abaixo da normalidade. A melhora foi mantida 5 meses após o término do tratamento.

Quanto à autopercepção de desempenho, o participante indicou melhora na habilidade de escrita, pois passou a fazer uso de lista de compras e registro de recados breves sobre atividades do cotidiano para a família imediata. Além disso, afirmou ter notado melhora na comunicação oral.

## DISCUSSÃO

O presente estudo evidenciou melhora clínica em atividade de escrita sob ditado, com estimulação das rotas fonológica e lexical por meio de terapia da linguagem escrita associada à ETCC em um idoso com afasia de condução e agrafia profunda. Os resultados sugerem melhora nas duas rotas, embora o efeito na rota fonológica tenha sido mais evidente e se mantido após 5 meses do tratamento. A melhora na escrita é relevante quando se considera o déficit crônico apresentado pelo participante em habilidades de emissão e compreensão de linguagem em testes padronizados (e.g., MTL).

O posicionamento do eletrodo positivo (em T5) foi guiado por simulação computacional (SimNIBS) de forma a maximizar o aumento de potencial elétrico sobre o córtex occipitotemporal esquerdo. Modelos recentes de processamento de linguagem escrita atribuem ao córtex occipitotemporal esquerdo papel importante na conversão fonema-grafema e no processamento lexical^(4)^. Ao estimular essa região, nosso objetivo foi potencializar o treinamento de conversão fonema-grafema e de acesso lexical.

O processamento via rota fonológica foi o que mais se beneficiou da terapia, conforme a resposta no ditado de pseudopalavras e palavras regulares de baixa frequência. Uma possibilidade é que esta melhora na escrita sob ditado tenha sido consequência da maior atuação do córtex occipitotemporal sob ETCC durante a conversão fonema-grafema, passo necessário para a escrita correta da palavra-alvo, via rota fonológica. Para as palavras irregulares, no entanto, é necessário o acesso à rota lexical, visto que requerem conhecimento prévio da ortografia, já que guiar-se apenas pela rota fonológica pode levar a erros de escrita. Além disso, apesar da queda no desempenho para palavras irregulares após 5 meses de intervenção, os resultados indicam melhora do processamento pela rota fonológica a longo prazo. Estudos com ETCC em indivíduos com afasia indicam que a aplicação cumulativa da estimulação pode ser necessária para se observar efeitos de longo prazo^(7)^.

No presente estudo, foram aplicadas apenas cinco sessões, suficientes para obtenção de benefício tardio. O caráter transitório do efeito observado em alguns testes (volta à linha de base 5 meses após o fim do tratamento) pode ser explicado pelo número de sessões. 

As evidências atuais mostram que o uso dessa ferramenta é bem tolerado por pessoas com quadros crônicos após acidente vascular cerebral e tem demonstrado benefícios terapêuticos para casos de afasia^(6,7)^. Quanto à generalização dos efeitos da intervenção, observou-se melhora considerável apenas para estímulos não treinados que dependem da rota fonológica.

O menor benefício na rota lexical e o retorno à pontuação de linha de base em determinados testes após um longo período do término da intervenção também podem ser explicados pelo nível de escolaridade e hábitos prévios de leitura, já que para o uso da rota lexical é necessário uso regular da ortografia.

O estudo apresenta algumas limitações referentes à avaliação inicial do participante, que não incluiu análises detalhadas das habilidades de entrada e saída de linguagem oral e escrita, o que poderia fornecer mais informações quanto ao benefício da ETCC no processamento da comunicação oral e gráfica. Os efeitos isolados de cada intervenção também não foram avaliados (i.e., somente treino de escrita vs. somente neuromodulação). Uma avaliação antes e após a intervenção com ETCC, sem concomitante treino de escrita, permitiria determinar mais precisamente o efeito isolado da ETCC no desempenho do participante. Além disso, não foram realizados testes padronizados logo após a intervenção com ETCC, o que não permitiu comparar o desempenho do paciente em testes padronizados antes e logo depois da intervenção com neuromodulação. Neste estudo, foram realizadas duas sessões quinzenais e três sessões intensivas em 3 dias da mesma semana. O espaçamento entre as sessões pode ter influenciado os resultados obtidos. Uma maior proximidade entre as sessões, principalmente as iniciais, poderia ter potencializado o desempenho do participante durante os testes aplicados. A partir da queixa inicial do participante, da melhora nos testes aplicados e do relato de melhora autopercebida, pode-se afirmar que houve melhora clínica e funcional na habilidade escrita. No entanto, ressalta-se cautela na interpretação dos resultados devido à pequena quantidade de itens avaliados. A aplicação de uma avaliação com maior quantidade de itens poderia ter proporcionado maior variabilidade de resposta.

## COMENTÁRIOS FINAIS

O treinamento de escrita sob ditado associado à ETCC sobre o córtex occipitotemporal esquerdo realizado com um participante com agrafia profunda proporcionou marcada melhora na avaliação clínica da emissão escrita de pseudopalavras e pequena melhora da escrita de palavras regulares de baixa frequência, ambos estímulos processados pela rota fonológica. Houve benefício imediato da estimulação na escrita de palavras irregulares, acessadas via rota lexical. O participante também apresentou pequena melhora para estímulos não treinados, sugerindo generalização. Os ganhos observados logo após o treinamento, no entanto, mantiveram-se apenas para a rota fonológica no ditado de acompanhamento realizado 5 meses depois. Os resultados são promissores dada a gravidade e cronicidade do caso e sugerem que a estimulação transcraniana por corrente contínua associada à terapia de escrita representa uma possível alternativa clínica para pacientes com agrafia profunda.
